# Supplementary material for: Activation of a Vibrio cholerae CBASS anti-phage system by quorum sensing and folate depletion
Source: mBio. 2023 Aug 25;14(5):e00875-23. doi: 10.1128/mbio.00875-23 (PMC10653837; doi:10.1128/mbio.00875-23)
Supplement: Supplemental Figures — Fig. S1-S4. [file mbio.00875-23-s0001.pdf]

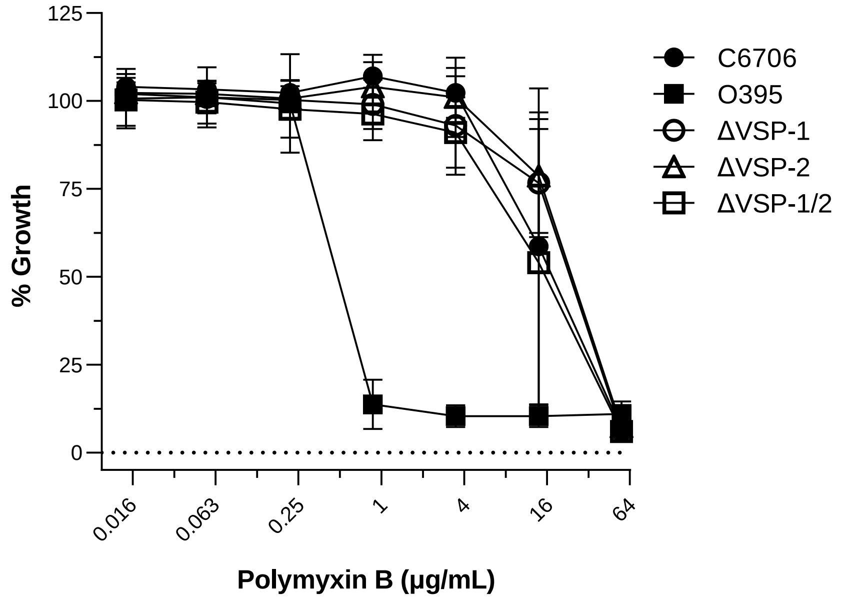

Figure S1: VSP-1 & -2 do not contribute to C6706 resistance to the antimicrobial peptide polymyxin B.

20-hour planktonic antibiotic sensitivity assay performed using a polymyxin B concentration gradient. % Growth reported as (OD_{600} polymyxin B treated / OD_{600} untreated) after 20 hours. Dotted line indicates 0% Growth. N = 3 biological replicates for all data points. Error bars represent standard deviation. IC_{50} for all strains are presented in Supplementary Table 1.

Fig. S1

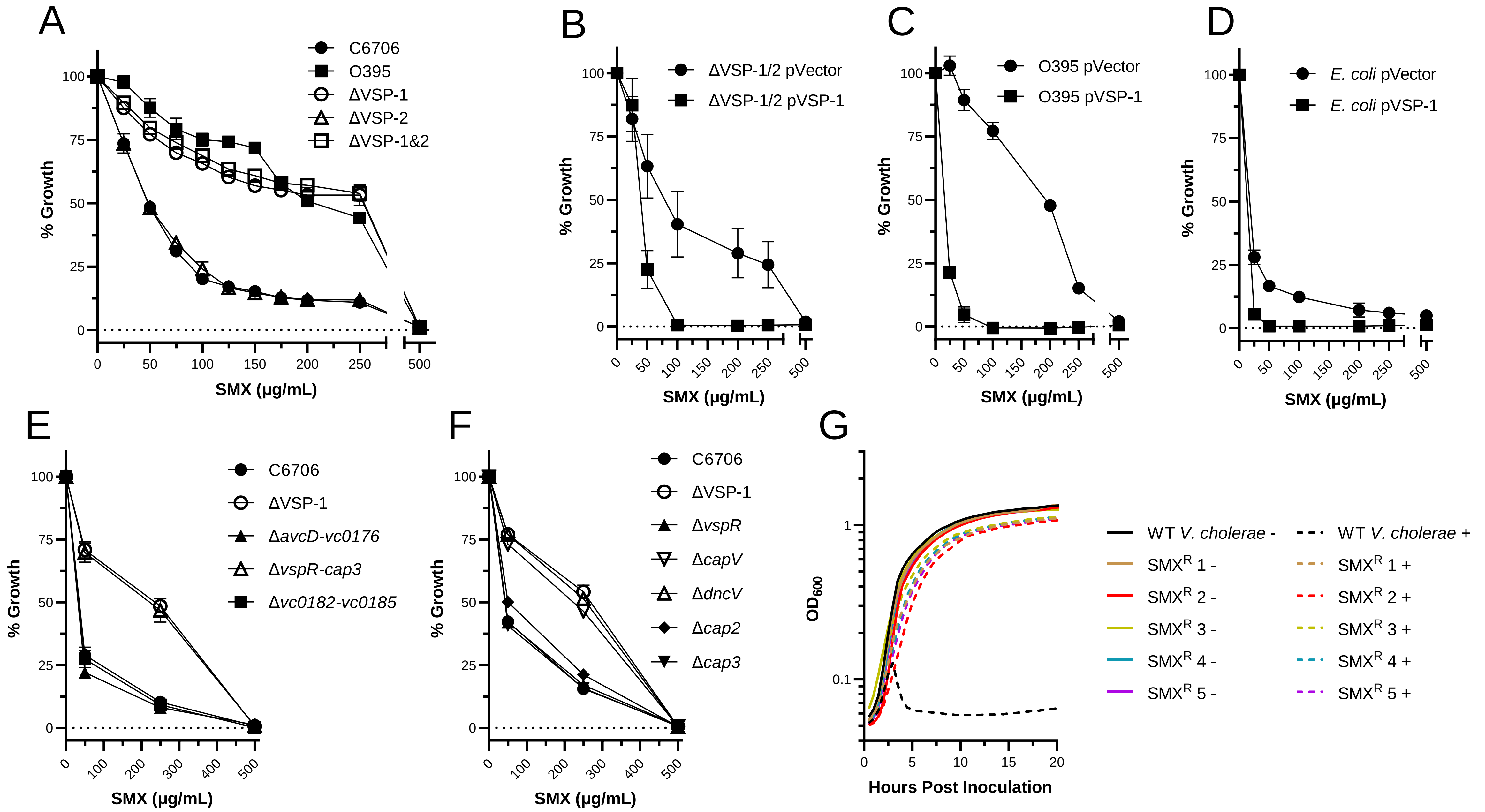
A
% Growth
SMX (µg/mL)
C6706
O395
ΔVSP-1
ΔVSP-2
ΔVSP-1&2
B
ΔVSP-1/2 pVector
ΔVSP-1/2 pVSP-1
C
O395 pVector
O395 pVSP-1
D
E. coli pVector
E. coli pVSP-1
E
C6706
ΔVSP-1
Δ*avcD-vc0176*
Δ*vspR-cap3*
Δ*vc0182-vc0185*
F
C6706
ΔVSP-1
Δ*vspR*
Δ*capV*
Δ*dncV*
Δ*cap2*
Δ*cap3*
G
OD_{600}
Hours Post Inoculation
WT *V. cholerae* -
WT *V. cholerae* +
SMX^R 1 -
SMX^R 1 +
SMX^R 2 -
SMX^R 2 +
SMX^R 3 -
SMX^R 3 +
SMX^R 4 -
SMX^R 4 +
SMX^R 5 -
SMX^R 5 +

Fig. S2

Figure S2: Scatter plots showing that VSP-1 encoded CBASS is responsible for *V. cholerae* biotype specific SMX sensitivity.

(**A-F**) 24-hour planktonic antibiotic sensitivity assays performed in a variety of SMX concentration gradients. Scatter plots represent the same data presented in heatmap form in (Figs. 2A, 2B, & 2D). N = 3 biological replicates and error bars represent standard error of the mean. IC_{50} for all strains in (**A**) are presented in Supplementary Table 1. (**G**) Growth curves of spontaneous SMX resistant isolates and the parental *V. cholerae* strain grown in LB with (+) and without (-) 50 µg/mL SMX. Lines depict the mean of triplicate cultures for each medium condition and indicated strain.

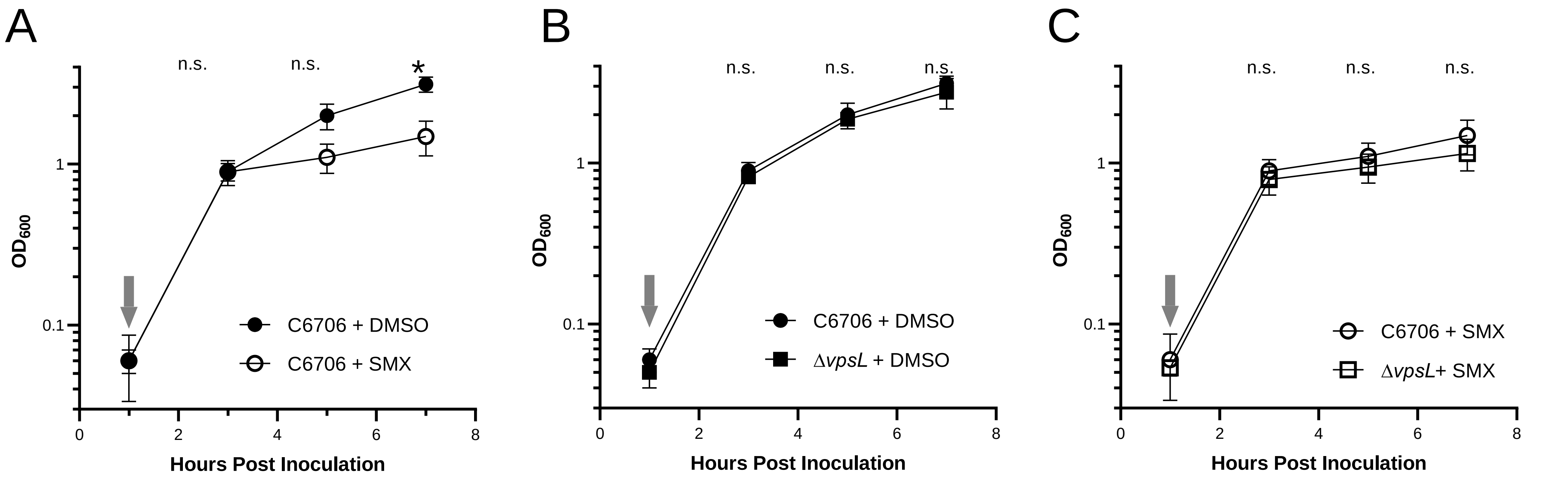

Figure S3: Lack of biofilm formation does not alter *V. cholerae* C6706 sensitivity to SMX.

Growth curves of (**A**) *V. cholerae* C6706 treated without (+ DMSO) and with 100 µg/mL SMX (+ SMX), and C6706 and Δ*vpsL* (**B**) untreated (+DMSO) and (**D**) treated (+SMX) with 100 µg/mL SMX. Grey arrows indicate addition of 100 µg/mL SMX or DMSO, approximately 1-hour after cultures were inoculated. N = 3 biological replicates and error bars represent standard deviation. For the purposes of statistical analysis, Δ*vpsL* data presented in (**B**) and (**C**) are also presented in (Fig. 3B). Statistical significance calculated using an unpaired *t* test with the Holm-Šídák method (*P < 0.05), n.s. = not significant.

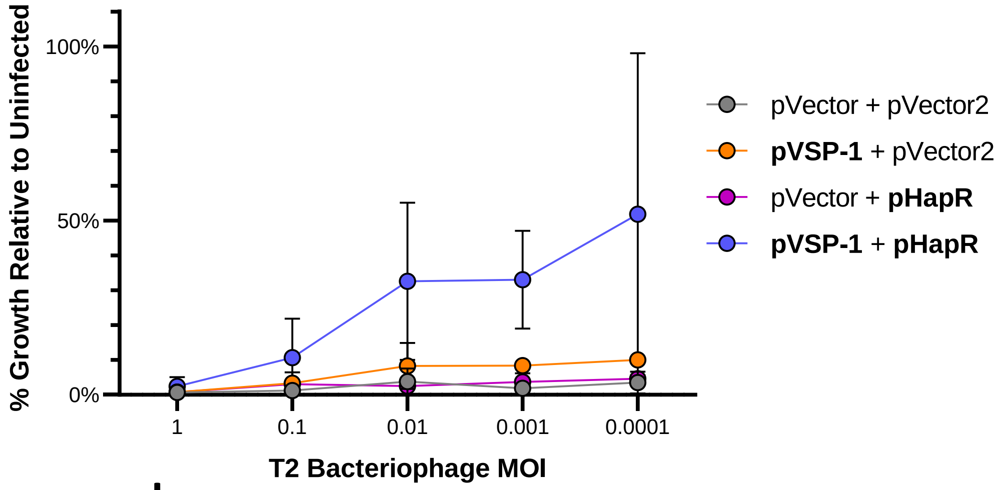

Figure S4: T2 phage infection graphs.

Growth of *E. coli* containing either pHapR induced with 10 μM IPTG and pVSP-1 with their associated vector controls after overnight growth with T2 phage. Data represent mean percent growth (OD_{600}) at the specified MOI calculated relative to control uninfected cultures. N = 3 biological replicates and error bars represent standard deviation. Data presented in heat map form in (Fig. 5A).

Fig. S4
