# Supplementary material for: Activation of a Vibrio cholerae CBASS anti-phage system by quorum sensing and folate depletion
Source: mBio. 2023 Aug 25;14(5):e00875-23. doi: 10.1128/mbio.00875-23 (PMC10653837; doi:10.1128/mbio.00875-23)
Supplement: Supplemental Tables — Tables S1-S5. [file mbio.00875-23-s0002.pdf]

Supplementary Table 1: *V. cholerae* Antibiotic Sensitivity

Strain	Antibiotic	IC_{50} (μg/mL)	R^2
C6706	Polymyxin B	18.4	0.77
O395	Polymyxin B	0.6	0.85
ΔVSP-1	Polymyxin B	22.1	0.84
ΔVSP-2	Polymyxin B	24.8	0.81
ΔVSP-1/2	Polymyxin B	14.5	0.84
C6706	Sulfamethoxazole	36.5	0.95
O395	Sulfamethoxazole	230.7	0.82
ΔVSP-1	Sulfamethoxazole	184.7	0.85
ΔVSP-2	Sulfamethoxazole	38.0	0.95
ΔVSP-1/2	Sulfamethoxazole	207.2	0.85

IC_{50} and R^2 calculated using a non-linear regression analysis of data presented in Fig. S1 (Polymyxin B) and Figs. 2A & S2A (Sulfamethoxazole)

Supplementary Table 2: Spontaneous SMX Resistance Isolate Genotype

SMX^R Isolate #	Mutation Description
1	*dncV* duplication 1192 to 1203, or 2872728 to 2872739 of chromosome
2	*capV* transposon insertion at position 597, or 2871046 of chromosome
3	*capV* transposon insertion at position 768, or 2871217 of chromosome
4	*dncV* 1 bp deletion at position 172, or 2871708 of chromosome
5	*dncV* transposon insertion at position 637, or 2872173 of chromosome

Variant calling was performed with BreSeq v.0.38.1 using default parameters against a reference genome (NCBI accession number: GCA_015482825.1)
The general location of each mutation is graphically depicted in Fig. 2C.

Supplementary Table 3. Bacterial strains used in this study.

Strains	Name in this Study	Relevant Characteristics	Source or reference
Escherichia coli			
BL21(DE3)	*E. coli* *(for SMX treatment, Figs. 2B & S3D)*	*F- ompT hsdSB(rB -mB +) gal dcm (DE3)*	Lab Stock
BW29427	-	*RP4-2(TetSkan1360::FRT)*, *thrB1004*, *lacZ58*(M15), *ΔdapA1341::[erm pir+]*, *rpsL*(strR), *thi-*, *hsdS-*, *pro-*	Lab Stock
DH10b	*E.coli* *(for luminescence and phage challenge assays, Figs. 4C & 5A)*	*F-mcrA Δ(mrr-hsdRMS-mcrBC) Φ80lacZΔM15 ΔlacX74 recA1 endA1 araD139Δ(ara, leu)7697 galU galK λrpsL nupG*	ThermoFisher Scientific
Vibrio cholerae			
O395	O395	Wild type O1 classical; Sm^R	DiRita Lab Stock (23)

C6706str2	WT C6706	Wild type O1 El Tor; Sm^R<br>-Quorum sensing proficient and does not contain the LuxO G333S quorum sensing variant found in some C6706 lineages (14)	Bassler Lab Lineage (8)
CR01	ΔVSP-1	C6706str2 ΔVSP-1	(8)
CR02	ΔVSP-2	C6706str2 ΔVSP-2	(8)
CR03	ΔVSP-1/2	C6706str2 ΔVSP-1/2	(8)
V. cholerae Δ*lacZ* (VC2338)	Δ*lacZ*	C6706str2 Δ*lacZ*	(59)
BYH255	Δ*acvD-vc0176*	C6706str2 Δ*acvD-vc0176*	(8)
BYH256	Δ*vspR-cap3*	C6706str2 Δ*vspR-cap3*	(8)
BYH257	Δ*vc0182-vc0185*	C6706str2 Δ*vc0182-vc0185*	(8)
GS07	Δ*vspR*	C6706str2 Δ*vspR* (not a deletion but a variant with nonsense mutations in codons 1, 2, and 6)	This study
WLN5105	Δ*capV*	C6706str2 Δ*capV*	(10)
GS01	Δ*dncV*	C6706str2 Δ*dncV*	(10)
WLN5380	Δ*cap2*	C6706str2 Δ*cap2*	(10)
WLN5381	Δ*cap3*	C6706str2 Δ*cap3*	(10)
CW2034	Δ*vpsL*	C6706str2 Δ*vpsL* biofilm-null	(35)
CW2036	Δ*vpsL*Δ*hapR*	C6706str2 Δ*vpsL* biofilm-null LCD-locked	(35)
CW2037	Δ*vpsL*Δ*luxO*	C6706str2 Δ*vpsL* biofilm-null HCD-locked	(35)
BH1842	*ΔcsqAΔluxS*	C6706str2 *ΔcsqAΔluxS*, LCD-locked with HCD induction by exogenous AIs	(37)
Phage			
T2	T2	Wild type	ATCC

Supplementary Table 4: Plasmids used in this study

Plasmids	Name in this Study	Relevant characteristics	Source or Reference
pLAFR	pVector	pLAFR cosmid; Tet^r	(64)
pCCD13	pVSP-1	pLAFR containing VSP-1; Tet^r	(10)
pBBR-lux	-		(57)
pKAD1	pP_{CBASS}::*lux*	pBBRlux containing 913bp 5' of *capV* locus (*i.e.,* CBASS promoter) to regulate luciferase expression	This study
pGBS54	-	pKAS32; Amp^r containing homologous region for introducing *vspR* nonsense mutations (creating strain GS07)	This study
pSLS13	pHapR	*hapR* allele cloned into pEVS143 under P_{tac}	(63)
pEVS141	pVector2	Promoterless pEVS143 vector control	(62)

Supplementary Table 5: Oligonucleotides used in this study

Name	Primer use	Sequence	Reference
Vector Construction Primers			
DS fwd VC0177 start stop	Construction of pGBS54	TAGTGACGTTCTATGTAGATCAGTGCAGAG	This study

DS rev VC0177 start stop	Construction of pGBS54	CTATAGTTCTAGAGGTACCTATGTCCTTTCAAAAAGCAAG	This study
US fwd VC0177 start stop	Construction of pGBS54	ATTCCCGGGAGAGCTGTGGTATGAAAGATGAGTGAAATAC	This study
US rev VC0177 start stop	Construction of pGBS54	CTACATAGAACGTCACTAGTATTTCTAATACCAC	This study
Lux_CBASSpr_FW	PCR P_{CBASS} region Rev (913 nt 5' *capV*) into pBBR-lux	CACCGCGGTGGCGGCCGCTCTAGAAAACGATATATAACCAAAGATCAAG	This study
Lux_CBASSpr_RV	PCR P_{CBASS} region Rev (913 nt 5' *capV* pBBR-lux	GGATTGAAAACGGAGGTTACCTCTAGTTGCGGCCGCAAAATGGAT	This study
RT-qPCR Primers			
CMW2926	*gyrA* F, housekeeping gene (Fig. 4A)	TGGCCAGCCAGAGATCAAG	(8)
CMW2927	*gyrA* R, housekeeping gene (Fig. 4A)	ACCCGCAGCGGTACGA	(8)
*capV*a F qPCR	*capV* F (Fig. 4A)	GTGCGCTTGGATAACAACATAC	This study
*capV*a R qPCR	*capV* R (Fig. 4A)	TACCGCGGCTAGCTAAATTAC	This study
*dncV*b F qPCR	*dncV* F (Fig. 4A)	ACAGACGAGGAGAAGAACAATC	This study
*dncV*b R qPCR	*dncV* R (Fig. 4A)	AGCCTTGGCAATACCCTTAG	This study
WNTP0011	*recA* F, housekeeping gene (Fig. 4B)	AATTTGTGATGCACTGGCTCGCTC	This study
WNTP0012	*recA* R, housekeeping gene (Fig. 4B)	CGTCAGTTTACGCATTGCTTGCGA	This study
WNTP1492	*capV* F (Fig. 4B)	CTTTGCAGATGGGGGATTGGTTGC	This study
WNTP1493	*capV* R (Fig. 4B)	CCTTTGCTTCAGGAAAGTCTGTTGCC	This study
WNTP1494	*dncV* F (Fig. 4B)	GCACCTTTCTGACAGCGAACAACG	This study
WNTP1495	*dncV* R (Fig. 4B)	GTCCAAAAGCGAGGTGTCAATCCC	This study
WNTP0015	*hapR* F (Fig. 4B)	CGTGACGAAGTTTGGCCACTGTTT	This study
WNTP0016	*hapR* R (Fig. 4B)	TGTTTCTCACACAATTCGCCACGC	This study
